# Preventive behavior against SARS-CoV-2 infection in adults according to whether or not they live with children. A combined analysis of the nationwide COSMO-SPAIN and ENE-COVID surveys

**DOI:** 10.3389/fpubh.2023.1061367

**Published:** 2023-02-23

**Authors:** Imane Jroundi, Nerea Fernández de Larrea-Baz, Carmen Rodríguez-Blázquez, Roberto Pastor-Barriuso, Marina Pollán-Santamaría, Maria João Forjaz, Beatriz Pérez-Gómez, the ENE-COVID Study Group, Pilar Aparicio Azcárraga

**Affiliations:** ^1^School of Medicine and Pharmacy of Rabat, Mohammed V University, Rabat, Morocco; ^2^National Centre for Epidemiology, Instituto de Salud Carlos III, Madrid, Spain; ^3^Consortium for Biomedical Research in Epidemiology and Public Health (CIBERESP), Madrid, Spain; ^4^Consortium for Biomedical Research in Neurodegenerative Diseases (CIBERNED), Madrid, Spain; ^5^The Health Services Research on Chronic Patients Network (REDISSEC) and Redes de Investigación Cooperativa Orientadas a Resultados en Salud (RICORS), Madrid, Spain

**Keywords:** COVID-19, pandemic, children's exposure, sociodemographic characteristics, prevention behaviors, health literacy, health knowledge, attitudes and practices

## Abstract

**Introduction:**

The protection of children is a major driver of behavior among those in charge of their care. We evaluated whether compliance with preventive measures against SARS-CoV-2 infection among adults living with children was different from that of those not living with them, in 2020.

**Methods:**

We used the COSMO-SPAIN (*N* = 867) and the nationally representative ENE-COVID (*N* = 29,926) surveys to estimate prevalence of compliance (95% confidence interval). Logistic model based standardization methods were applied to estimate standardized prevalence differences (SPrD) to the overall distribution of age, sex, education, history of COVID-19, and residence of other >60 yrs in the household.

**Results:**

We observed that adults living with children more frequently avoided bars (SPrD_ENE−COVID_: 4.2%; 95% CI: 2.3–6.1), crowded places (SPrD_COSMO_: 8.0%; 95% CI: 0.6–15.1) and did not use public transportation (SPrD_ENE−COVID_: 4.9%; 95% CI: 3.0–6.7). They were also more worried about work and family conciliation (SPrD_COSMO_: 12.2%; 95% CI: 4.8–19.5) and about closure of education centers (SPrD_COSMO_: 26.5%; 95% CI: 19.4–33.6).

**Discussion:**

In general, adults living with children adopted slightly more frequently social distancing measures.

## 1. Introduction

During the first waves of the COVID-19 pandemic, adults living with children faced particular difficulties and worries (1–3). On the one hand, they were concerned about children's health, mainly at the beginning of the pandemic, when there was still high uncertainty about the severity of the infection among them (4). On the other hand, they had specific worries related to the consequences that some public health control measures, such as lock-downs, social distance recommendations (5) or school closure (6) could have on children' physical and mental health (7), and on their development and education (8).

All these aspects may have influenced the attitudes and practices of this population group during the pandemic (2). They may have been more interested in learning about the COVID-19, its transmission mechanisms, and preventive measures, and, perhaps, they may have been more compliant with preventive measures, both to protect and to serve as an example for their children, as has been observed in other emergencies (9). It is known that parents are important socialization agents who play the role of health promoters, role models, and educators in the lives of their children (10) and, in this case, they were responsible for teaching and making their children follow the preventive measures established to reduce the risk of getting SARS-CoV-2 infection, modulating this way the capacity of protecting themselves. On the other hand, they might have been more flexible in the implementation of some measures in order to avoid potentially negative consequences of certain preventive recommendations (e.g., confinement), especially when the epidemiologic data showed that children's infection was usually mild.

According to the conceptual framework of knowledge, attitudes and practices (KAP), behaviors are influenced by knowledge about their benefits and risks, together with attitudes related to them (11). The study of these aspects in parents and other adults living with children during the pandemic can help to understand how did they live through this unique situation, and possible reasons for different preventive behaviors. It may also provide clues to evaluate the success of the information campaigns and to improve health promotion in the future in this subgroup of the population.

In the present work, we took advantage of the nationwide COSMO-SPAIN (12) and ENE-COVID (13) surveys to test the hypothesis that young and middle-aged adults living with children differ in their preventive behavior, knowledge, perception of risk, and concerns related to the COVID-19 compared to those who did not live with them. COSMO-SPAIN, the World Health Organization (WHO) Behavioral Insights survey on COVID-19 in Spain (14) is a nationwide repeated online survey designed to collect data on KAP and risk perceptions related to this disease in adult population. As for ENE-COVID (13), it is a nationwide population-based sero-epidemiological survey of SARS-CoV-2 infection in Spain with more than 50,000 participants, representative of the general population living in households in the country, which collected information on behavior related to control or reduce exposure to this virus. Both, COSMO-SPAIN and ENE-COVID had several rounds. For the present study, we will focus on data extracted from their third and fourth rounds (15, 16) respectively, which took place during November 2020.

## 2. Materials and methods

### 2.1. Design

COSMO-SPAIN, the World Health Organization (WHO) Behavioral Insights survey on COVID-19 in Spain (12, 14), is coordinated by the Carlos III Health Institute, with the aim of monitoring the behavior and attitudes of the population related to COVID-19 in the country. It consisted on a nationwide, cross sectional panel survey whose field work was entrusted to a consumer research company. In each round, people aged 18 years or older were invited by email to answer an online questionnaire, until gathering a sample that matched the distribution of sociodemographic characteristics of the Spanish general population (age, education, gender and large area of residence). In the 3rd round, 2,655 people residing in Spain were invited to participate, of which 1,777 responded and 1,018 completed the questionnaire on time (15).

The ENE-COVID survey was developed and driven by the Carlos III Health Institute, the Spanish Ministry of Health, the Spanish Institute of Statistics and the Health Services of all the regions in Spain (13). Its aims were to investigate the prevalence of SARS-CoV-2 infection in the non-institutionalized population in Spain, overall and at province level, by testing antibodies against the virus and exploring their temporal evolution, and to evaluate factors related to infection. A random sample of 35,883 households was initially selected through a two-stage stratified sampling, with strata formed by province and municipality size. All residents in each household were invited to participate. In the 4th round of the survey, a total of 51,409 subjects participated (54.7% of those eligible). Candidates were invited by phone, and those who accepted were scheduled for a visit in a healthcare center or in their own house. All the participants were tested for SARS-CoV-2 antibodies and answered an epidemiological questionnaire (by phone or in a face-to-face interview). A common training platform was developed for collaborators in the data collection process, and continued contact with the study organizations was allowed to solve possible doubts and homogenize procedures.

Both surveys included information about household composition, allowing identifying those in which children lived. The design of both studies has been previously described in detail (13, 15).

Protocols of the ENE-COVID and COSMO-SPAIN studies (available at https://repisalud.isciii.es/handle/20.500.12105/15247 and https://doi.org/10.23668/psycharchives.4877, respectively), were reviewed and approved by the ethics committee of Carlos III Institute of Health, and participants provided written informed consent to participate.

### 2.2. Study population

For the present study, all the adults aged 25 to 64 years old participating in the third round of the COSMO-SPAIN and the fourth round of the ENE-COVID studies, both of them carried out during November 2020, were selected and classified as living with children or not living with children younger than 14 years.

#### 2.2.1. Epidemiological context during the field work of the studies

The 3rd round of the COSMO-SPAIN survey was carried out between November 24 and 27, 2020 and the 4th round of the ENE-COVID from November 16 to 29, 2020. During this period, the 14 days cumulative incidence of COVID-19 in Spain was around 307 cases/100,000 habitants (17) and global national seroprevalence (considering the positives at any time up to November 2020) was estimated as 9.9% (95% CI: 9.4–10.4) (16).

During those weeks, inter-regions mobility restrictions, and schedule and capacity limitations were maintained in commercial establishments in several regions, while the schools remained open since September 2020. Facemask use was mandatory for people aged 6 years or older in all public spaces where a distance of 1.5 meters could not be guaranteed, including in the outdoors, and in transports.

### 2.3. Variables of interest

The epidemiological questionnaires of both, COSMO-SPAIN and ENE-COVID studies, included information about some sociodemographic and COVID-19 related variables, such as employment situation, education level and COVID-19 testing.

#### 2.3.1. Compliance with preventive measures

In the COSMO-SPAIN study, preventive behaviors were assessed through the question “During the last seven days, how frequently did you take the following measures to prevent infection from coronavirus/COVID-19?”, with five answer options from 1 (never) to 5 (always). Preventive measures related to mask using, hand washing, ventilating indoor places, disinfecting surfaces, physical distancing and social distancing were included in the questionnaire. For the present work only those related to aspect also addressed in the ENE-COVID survey were analyzed: wearing mask according to recommendations, wearing a mask when being with friends, type of mask, ventilating closed spaces, avoiding crowded places, avoiding public transport, maintaining physical distance (at least 2 meters), and avoiding social/family meetings.

In the ENE-COVID study, information about preventive measures was gathered using questions about frequency of doing certain activities or preventive actions during the last 5 months (from July to November 2020), e.g. “Since the first of July 2020… Do you wear a mask during meetings with family or friends (in your home or in theirs)?” The preventive measures included were: using public transport, wearing mask in different settings (at work/school, during displacements, in meetings with family or friends, and in other situations with people not living in the same household), type of mask, ventilating in the work place, maintaining physical distance at work (at least 1.5 meters), going out for a drink or lunch (indoor and outdoor) and attendance to events with more than 10 people (indoor or outdoor). Answer options varied depending on the question (e.g., Yes/No or Yes/No/Sometimes).

Some behaviors were only explored in one of the surveys. For those preventive behaviors included in both questionnaires, we recodified the variables to make them comparable. We classified answers “yes” and “always” options as “yes”, and the other options, i.e., “no”, “sometimes”, “not always” or “do not know” as “no”.

#### 2.3.2. Knowledge, risk perception, and level of worry

Information regarding these aspects was only collected in the COSMO-SPAIN study. Knowledge about COVID-19 and its prevention was assessed asking about the correctness of several statements (response options: “yes”, “no”, or “do not know”). Risk perception was assessed by asking about the probability of getting infected in several settings, in a scale of 1 (very unlikely) to 5 (very likely), and about the severity of the disease if they were infected, in a scale from 1 (not severe) to 5 (very severe).

To assess the level of worry about several possible consequences of the pandemic, the question used was “At the moment, how much do you worry about . . .?” and the answer options ranged from 1 (do not worry at all) to 5 (worry a lot). For the present work, all these variables were also dichotomized, grouping the two highest answers (e.g., 4 “worry” and 5 “worry a lot”) in one category and the other three in another category. Information regarding worries was only collected in the COSMO-SPAIN study.

#### 2.3.3. Personal history of COVID-19

In the ENE-COVID survey, both, self-reported information on PCR or antigen test results, pneumonia or hospitalization due to COVID-19, and the result of the serology test applied within the framework of the study were considered. In the COSMO-SPAIN survey, self-reported positivity to diagnostic test was collected.

**Figure 1:**
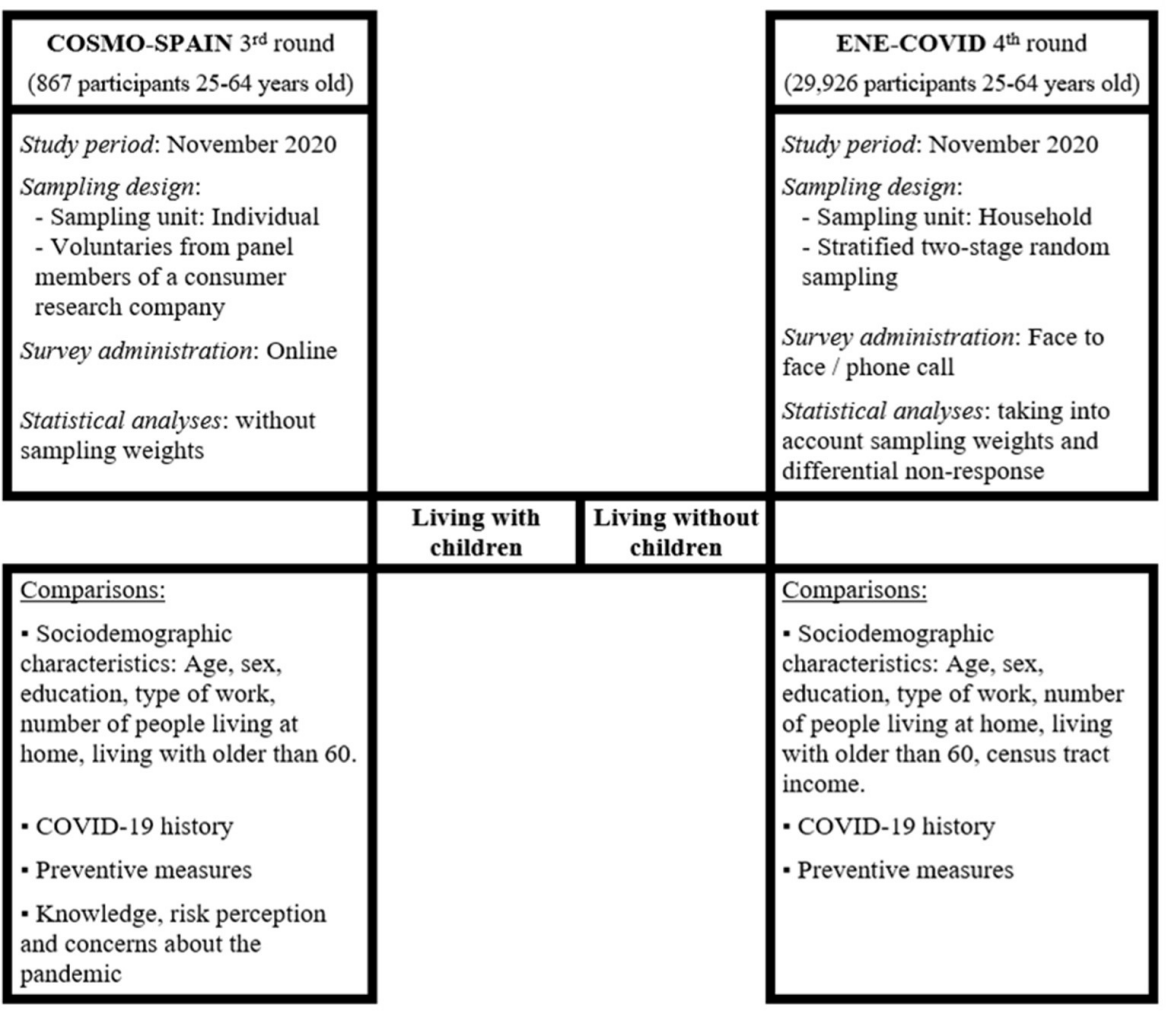
Overview of the current study design based on data from COSMO-SPAIN and ENE-COVID, November 2020.

Figure 1 shows an overview of the present study design based on data from COSMO-SPAIN and ENE-COVID.

### 2.4. Statistical analyses

For the ENE-COVID survey, sampling weights were assigned to study participants to account for the different sampling selection probabilities and to adjust for non-response based on sex, age, and average relative income in the census tract. Confidence intervals were estimated taking into account stratification by province and municipality size and the clustering by household and census tract (13, 18).

As descriptive statistics, frequencies and percentages were calculated for sociodemographic and COVID-19 related variables, both in overall population samples and in the groups of participants living or not living with children in COSMO-SPAIN and ENE-COVID surveys. Also, we described adherence to COVID-19 preventive recommendations according to socioeconomic characteristics and COVID-19 experience in both studies.

Afterwards, we estimated prevalences and 95% confidence intervals (CIs) of compliance with preventive measures, as well as of knowledge, risk perception, and worry on COVID-19, for participants living or not living with children. To adjust for confounding by sociodemographic and COVID-19 related characteristics, prevalence differences for each outcome by presence of children in the household were standardized to the overall distribution of age, sex, educational level, living with someone older than 60, and personal history of COVID-19 in the entire population by using logistic model-based standardization methods (19). These standardized prevalence differences (SPrD) represent the differences in the outcome prevalence that would have been observed between people living with and without children, had both groups had the same distribution of confounding factors as the overall population. We used standardized prevalence differences as absolute effect measures because they are more informative and readily interpretable than relative effect measures, such as prevalence ratios and odds ratios, when the prevalence is high, as it is the case for most outcome variables in this study.

Analyses were carried out in Stata, version 16 (StataCorp. 2019. Stata Statistical Software: Release 16. College Station, TX: StataCorp LLC) and SPSS 27© (IBM Corp., Armonk, NY, USA).

## 3. Results

### 3.1. Sociodemographic characteristics

The general characteristics of the adults aged 25 to 64 years who participated in the 4th round of the ENE-COVID (*N* = 29,926) and in the 3rd round of the COSMO-SPAIN (*N* = 867), by presence of children <14 years old in the home are described in Table 1.

**Table 1:** General characteristics of COSMO-SPAIN and ENE-COVID participants aged 25–64 years according to the presence of children in the household, November 2020.

	**COSMO-SPAIN**			**ENE-COVID**		
	**Total** ***n* (%)**	**Living with children** ***n* (%)**	**Not living with children** ***n* (%)**	**Total** ***n* (%)**	**Living with children** ***n* (%)**	**Not living with children** ***n* (%)**
*Total*	*867 (100%)*	*258 (29.8%)*	*609 (70.2%)*	*29,926 (100%)*	*9,107 (30.4%)*	*20,819 (69.6%)*
**Variable**						
**Sex**						
Men	407 (46.9%)	125 (48.5%)	282 (46.3%)	13,831 (46.2%)	4,081 (44.8%)	9,750 (46.8%)
Women	460 (53.1%)	133 (51.5%)	327 (53.7%)	16,095 (53.8%)	5,026 (55.2%)	11,069 (53.2%)
**Age group (years)**						
25-34	217 (25.0%)	42 (16.3%)	175 (28.7%)	4,312 (14.4%)	1,106 (12.1%)	3,206 (15.4%)
35-44	230 (26.5%)	106 (41.1%)	124 (20.4%)	7,425 (24.8%)	4,446 (48.8%)	2,979 (14.3%)
45-54	242 (27.9%)	89 (34.5%)	153 (25.1%)	9,210 (30.8%)	2,946 (32.3%)	6,264 (30.1%)
55-64	178 (20.5%)	21 (8.1%)	157 (25.8%)	8,979 (30.0%)	609 (6.7%)	8,370 (40.2%)
**Education level**						
Primary or less	202 (23.3%)	58 (22.5%)	144 (23.6%)	10,955 (37.0%)	2,879 (31.9%)	8,076 (39.2%)
Secondary	276 (31.8%)	86 (33.3%)	190 (31.2%)	10,492 (35.4%)	3,346 (37.1%)	7,146 (34.7%)
University	389 (44.9%)	114 (44.2%)	275 (45.2%)	8,192 (27.6%)	2,794 (31.0%)	5,398 (26.2%)
**Number of persons living in the house**						
1	98 (11.3%)	0 (0.0%)	98 (16.1%)	1,910 (6.4%)	0 (0.0%)	1,910 (9.2%)
2	275 (31.7%)	17 (6.6%)	258 (42.4%)	7,029 (23.5%)	161 (1.8%)	6,868 (33.0%)
3	216 (24.9%)	85 (33.0%)	131 (21.5%)	8,472 (28.3%)	2,517 (27.6%)	5,955 (28.6%)
4	185 (21.3%)	88 (34.1%)	97 (15.9%)	8,415 (28.1%)	4,016 (44.1%)	4,399 (21.1%)
≥5	93 (10.7%)	68 (26.4%)	25 (4.1%)	4,100 (13.7%)	2,413 (26.5%)	1,687 (8.1%)
**Living with older than 60 years**						
No	699 (80.6%)	225 (87.2%)	474 (77.8%)	21,276 (71.1%)	7,778 (85.4%)	13,498 (64.8%)
Yes	168 (19.4%)	33 (12.8%)	135 (22.2%)	8,648 (28.9%)	1,327 (14.6%)	7,321 (35.2%)
**Employed**						
No	300 (34.6%)	78 (30.2%)	222 (36.5%)	9,902 (33.1%)	2,338 (25.7%)	7,564 (36.3%)
Yes	567 (65.4%)	180 (69.8%)	387 (63.5%)	20,024 (66.9%)	6,769 (74.3%)	13,255 (63.7%)
**Health care worker**						
No	546 (96.3%)	173 (96.1%)	373 (96.4%)	18,885 (94.3%)	6,393 (94.4%)	12,492 (94.2%)
Yes	21 (3.7%)	7 (3.9%)	14 (3.6%)	1,139 (5.7%)	376 (5.6%)	763 (5.8%)
**Telework**						
No	466 (82.2%)	150 (83.3%)	316 (81.7%)	18,372 (91.8%)	6,197 (91.6%)	12,175 (91.8%)
Yes	101 (17.8%)	30 (16.7%)	71 (18.3%)	1,652 (8.3%)	572 (8.4%)	1,080 (8.1%)
**Municipality size (habitants)**	-	-	-			
>100,000				9,006 (30.1%)	2,647 (29.1%)	6,359 (30.5%)
20,000–100,000				8,973 (30.0%)	2,969 (32.6%)	6,004 (28.8%)
5,000–20,000				6,515 (21.8%)	2,047 (22.5%)	4,468 (21.5%)
< 5,000				5,432 (18.1%)	1,444 (15.9%)	3,988 (19.2%)
**Census tract average income**	-	-	-			
> 17,000 Euros				1,613 (5.4%)	457 (5.0%)	1,156 (5.6%)
12,500–17,000 Euros				6,432 (21.5%)	1,869 (20.5%)	4,563 (21.9%)
9,000-12,500 Euros				14,480 (48.4%)	4,351 (47.8%)	10,129 (48.7%)
7,700-9,000 Euros				4,665 (15.6%)	1,524 (16.7%)	3,141 (15.1%)
7,100-7,700 Euros				1,404 (4.7%)	420 (4.6%)	984 (4.7%)
< 7,100 Euros				1,332 (4.4%)	486 (5.3%)	846 (4.1%)
**Regional seroprevalence**						
< 6.5%	397 (45.8%)	116 (45.0%)	281 (46.1%)	8,387 (28.0%)	2,492 (27.4%)	5,895 (28.3%)
6.5–9%	72 (8.3%)	21 (8.1%)	51 (8.4%)	8,115 (27.1%)	2,464 (27.1%)	5,651 (27.1%)
9–12%	196 (22.6%)	52 (20.2%)	144 (23.6%)	5,097 (17.0%)	1,688 (18.5%)	3,409 (16.4%)
≥12%	202 (23.3%)	69 (26.7%)	133 (21.8%)	8,327 (27.8%)	2,463 (27.0%)	5,864 (28.2%)

Dashes indicate information not available in the corresponding study.

Among COSMO-SPAIN participants, 258 (29.8%) were living with children at the time of the survey. Overall, 460 (53.1%) were women, 389 (44.9%) had university education, and 101 (17.8%) worked remotely. In the ENE-COVID survey, 9,107 participants (30.4%) were living with children. Regarding education, 8,192 (27.6%) had a university level, and 1,652 (8.3%) worked remotely. In both studies, participants not living with children were older, and more frequently lived with someone older than 60.

### 3.2. Personal history of COVID-19

In COSMO-SPAIN study, 69 (7.9%) participants reported having had COVID-19, which was severe in 8 (0.9%) of them. In the ENE-COVID study, 4,261 participants (14.2%) had been infected, while 634 (2.1%) reported having had pneumonia or hospital admission due to COVID-19. In both studies, the distribution of COVID-19 related variables in participants living with children was similar to that of participants not living with children, with no relevant differences in the magnitude of the percentages (Table 2).

**Table 2:** COVID-19 history of COSMO-SPAIN and ENE-COVID participants aged 25–64 years, according to the presence of children in the household, November 2020.

	**COSMO-SPAIN**			**ENE-COVID**		
	**Total**	**Living with children**	**Not living with children**	**Total**	**Living with children**	**Not living with children**
**Variable**	***n*** **(%)**	***n*** **(%)**	***n*** **(%)**	***n*** **(%)**	***n*** **(%)**	***n*** **(%)**
**Personal history of COVID-19**						
No infection	798 (92.0%)	234 (90.7%)	564 (92.6%)	25,665 (85.8%)	7,832 (86.0%)	17,833 (85.7%)
Not severe-infection	61 (7.0%)	21 (8.1%)	40 (6.6%)	3,627 (12.1%)	1,088 (11.9%)	2,539 (12.2%)
Pneumonia or hospitalization	8 (0.9%)	3 (1.2%)	5 (0.8%)	634 (2.1%)	187 (2.0%)	447 (2.2%)
**Self-reported positive PCR/antigen test**						
No	818 (94.3%)	237 (91.9%)	581 (95.4%)	28,551 (95.4%)	8,675 (95.3%)	19,876 (95.5%)
Yes	49 (5.7%)	21 (8.1%)	28 (4.6%)	1,374 (4.6%)	432 (4.7%)	942 (4.5%)
**Positive IgG antibody testing (in the 4**^th^ **round)**						
No	-	-	-	27,753 (92.7%)	8,476 (93.1%)	19,277 (92.6%)
Yes	-	-	-	2,172 (7.3%)	631 (6.9%)	1,541 (7.4%)
**COVID-19 at home**						
No	-	-	-	25,799 (86.2%)	7,807 (85.7%)	17,992 (86.4%)
Yes	-	-	-	4,127 (13.8%)	1,300 (14.3%)	2,827 (13.6%)
**A close friend or relative has or had COVID-19**						
No	353 (40.7%)	103 (39.9%)	250 (41.0%)	-	-	-
Yes	514 (59.3%)	155 (60.1%)	359 (59.0%)	-	-	-
**Contact with a confirmed case during the last month**						
No	-	-	-	27,723 (92.6%)	8,329 (91.5%)	19,394 (93.2%)
Yes	-	-	-	2,203 (7.4%)	778 (8.5%)	1,425 (6.8%)

Dashes indicate information not available in the corresponding study.

### 3.3. Preventive measures

Adherence to preventive measures of COSMO-SPAIN and ENE-COVID participants according to sociodemographic characteristics and COVID-19 experience is described in Supplementary Tables 1, 2. The factors most consistently associated with preventive behaviors in both studies were sex, age, education (probably reflecting the age structure), and being a healthcare worker. Additionally, in the ENE-COVID study, participants living in areas with lower average income, reported higher adherence to preventive measures, except for the avoidance of visiting family.

Table 3 presents standardized prevalence differences between those living with children and those who did not live with them, after adjusting for age, sex, education level, living with older people and personal history of COVID-19. Our results showed a better compliance with mask wearing in general (SPrDCosmo: 6.2%; 95% CI: 0.4–11.9) among people living with children. In regard to mask use in family meetings/with friends, results were not consistent among our two studies - COSMO-Spain (SPrDCosmo: 5.5%; 95% CI: −1.6–12.6) vs ENE-COVID (SPrDEnecovid: −1.1%; 95% CI: −3.5–1.4). On the other hand, in the ENE-COVID study, use of masks during leisure time was slightly more common among those living with children. Regarding type of mask, ENE-COVID results showed that individuals living with children tended to use hygienic mask more than those who didn't, and the opposite was observed for FFP2 masks.

**Table 3:** Preventive practices of young and middle-aged COSMO-SPAIN and ENE-COVID participants, according to the presence of children in the household, November 2020.

	**COSMO–SPAIN**					**ENE–COVID**				
	**Living with children**		**Not Living with children**		**Standardized prevalence difference^a^, % (95% CI)**	**Living with children**		**Not living with children**		**Standardized prevalence difference^a^, % (95% CI)**
**Preventive measures** ^c^	***n***	**% (95% CI)**	***n***	**% (95% CI)**		***n***	**% (95% CI)** ^b^	***n***	**% (95% CI)** ^b^	
**Always wearing face masks**										
In general	213	82.6 (77.4–86.7)	474	77.8 (74.4–81.0)	6.2 (0.4 to 11.9)	–	–	–	–	
In family meetings/with friends	166	64.3 (58.3–70.0)	366	60.1 (56.1–63.9)	5.5 (−1.6 to 12.6)	3,558	41.0 (38.8–43.1)	8,317	43.6 (42.0–45.1)	−1.1 (−3.5 to 1.4)
In the working place	–	–	–	–		6,256	92.8 (91.8–93.7)	12,201	90.9 (90.2–91.6)	1.8 (0.6 to 3.0)
During leisure time	–	–	–	–		8,731	97.5 (97.0–98.0)	19,721	96.6 (96.2–97.0)	1.0 (0.4 to 1.6)
During displacements	–	–	–	–		8,904	98.3 (97.9–98.6)	20,291	98.2 (97.9–98.5)	0.0 (−0.5 to 0.5)
**Type of mask**										
Hygienic	43	18.6 (14.1–24.2)	114	21.3 (18.0–24.9)	−3.4 (−9.7 to 2.9)	1,894	22.7 (21.2–24.2)	3,474	18.6 (17.7–19.5)	1.9 (0.2 to 3.6)
Surgical	118	51.1 (44.6–57.5)	262	48.9 (44.7–53.1)	2.7 (−5.4 to 10.7)	5,458	59.1 (57.4–60.8)	12,931	60.7 (59.6–61.8)	0.7 (−1.4 to 2.8)
FFP2	70	30.3 (24.7–36.5)	160	29.9 (26.1–33.9)	0.7 (−6.7 to 8.1)	1,743	18.2 (17.0–19.5)	4,391	20.8 (19.9–21.7)	−2.6 (−4.3 to −1.0)
**Maintaining physical distance**										
In general	111	43.0 (37.1–49.1)	253	41.5 (37.7–45.5)	1.6 (−5.8 to 9.0)	–	–	–	–	
At work	–	–	–	–		5,644	84.1 (82.8–85.4)	11,284	83.3 (82.3–84.2)	1.9 (0.2 to 3.5)
**Social distance**										
Avoid family meetings	99	38.4 (32.6–44.5)	254	41.7 (37.8–45.7)	−2.4 (−9.7 to 4.8)	1,250	13.1 (12.0–14.3)	3,075	13.3 (12.6–14.1)	1.2 (−0.2 to 2.6)
Avoid crowded places	149	57.8 (51.6–63.6)	311	51.1 (47.1–55.0)	8.0 (0.6 to 15.5)	–	–	–	–	
Avoid social events with >10 people	–	–	–	–		5,988	64.9 (63.1–66.5)	15,527	73.2 (72.0–74.4)	−5.8 (−7.8 to −3.9)
Avoid going inside bars	–	–	–	–		6,589	71.1 (69.5–72.7)	14,251	67.6 (66.5–68.7)	4.2 (2.3 to 6.1)
Avoid going outside bars	–	–	–	–		3,441	35.3 (33.6–37.0)	7,645	34.5 (33.4–35.6)	3.8 (1.6 to 5.9)
**Ventilating spaces**										
Closed spaces in general	159	61.6 (55.5–67.4)	404	66.3 (62.5–70.0)	−5.1 (−12.3 to 2.1)	–	–	–	–	
Working place	–	–	–	–		6,137	90.6 (89.4–91.7)	12,478	91.1 (90.3–91.8)	0.3 (−1.1 to 1.7)
**Public transport**										
Not using public transport	–	–	–	–		7,271	77.3 (75.8–78.8)	15,804	72.3 (71.2–73.4)	4.9 (3.0 to 6.7)
Avoid the use of public transport	167	64.7 (58.7–70.3)	380	62.4 (58.5–66.2)	1.6 (−5.8 to 8.9)	–	–	–	–	

a Standardized to the overall distribution of sex, age, education, living with someone older than 60, and personal history of COVID-19 in the entire COSMO-SPAIN or ENE-COVID population.

b Prevalences and 95% confidence intervals (CIs) were estimated accounting for sampling weights and design effects of stratification and clustering in ENE-COVID survey.

c Wording of the corresponding items in the questionnaires is available in the Supplementary material.

With respect to social distancing, according to the COSMO-SPAIN study, 57.8% (95% CI: 51.6–63.6) participants living with children avoided going to crowded places, compared to 51.1% (95% CI: 47.1–55.0) of those not living with children (SPrD: 8.0%; 95% CI: 0.6–15.5). The ENE-COVID results also showed higher social distance measures among people living with children, as reflected by a higher frequency of not going to bars, either inside (SPrD: 4.2%; 95% CI: 2.3–6.1) or outside (SPrD: 3.8%; 95% CI: 1.6–5.9), and not using public transportation (SPrD: 4.9%; 95% CI: 3.0–6.7).

Only ENE-COVID study explored behavior in the working place. A slightly higher proportion of people living with children reported observing physical distance (SPrD: 1.9%; 95% CI: 0.2–3.5) and wearing mask at work (SPrD: 1.8%; 95% CI: 0.6–3.0) (Table 3).

### 3.4. Knowledge, risk perception and level of worry

The percentage of right answers to questions exploring knowledge on COVID-19 mode of transmission and preventive measures was very high (ranging from 85.5 to 98.8%). The lowest percentages corresponded to the understanding of the latency period (85.9% chose the right answer), and to the consideration of authority recommendations as mandatory, which was correctly answered by 85.5% of the sample. No noteworthy differences were observed between adults living with children and those without children at home, except for a slightly higher proportion of correct answers in the questions related to hand washing (SPrD: 2.3%; 95% CI: −0.7 to 5.4) and maintaining physical distance (SPrD: 2.3%; 95% CI: −0.7 to 5.3), among those living with children (Table 4).

**Table 4:** Knowledge, risk perception and worries on COVID-19 in the COSMO-SPAIN participants, according to presence of children in the household, November 2020.

	**Total**		**Living with children**		**Not living with children**		**Standardized prevalence difference^e^, % (95% CI)**
	***N***	**% (95% CI)**	***N***	**% (95% CI)**	***N***	**% (95% CI)**	
**Knowledge** ^a^							
COVID−19 is spread by drops when coughing/talking	831	95.8 (94.3–97.0)	246	95.3 (92.0–97.3)	585	96.1 (94.2–97.3)	−0.1 (−3.0 to 2.8)
People who don't have fever can be contagious	805	92.8 (90.9–94.4)	242	93.8 (90.1–96.2)	563	92.4 (90.1–94.3)	0.8 (−3.1 to 4.6)
COVID−19 symptoms appear as soon as you get infected	745	85.9 (83.4–88.1)	226	87.6 (83.0–91.1)	519	85.2 (82.2–87.8)	2.4 (−2.6 to 7.4)
The recommendations of the authority are mandatory	741	85.5 (83.0–87.7)	224	86.8 (82.1–90.4)	517	84.9 (81.8–87.5)	2.8 (−2.3 to 7.9)
If I have symptoms I should stay at home	848	97.8 (96.6–98.6)	251	97.3 (94.4–98.7)	597	98.0 (96.6–98.9)	0.2 (−1.8 to 2.3)
If I am a close contact I must isolate myself	843	97.2 (95.9–98.1)	251	97.3 (94.4–98.7)	592	97.2 (95.6–98.3)	0.9 (−1.3 to 3.2)
Face masks should cover mouth and nose	857	98.8 (97.9–99.4)	256	99.2 (96.9–99.8)	601	98.7 (97.4–99.3)	1.0 (−0.4 to 2.3)
Hands should be washed before and after using the face mask	821	94.7 (93.0–96.0)	248	96.1 (92.9–97.9)	573	94.1 (91.9–95.7)	2.3 (−0.7 to 5.4)
Maintaining physical distance is an effective measure	821	94.7 (93.0–96.0)	247	95.7 (92.5–97.6)	574	94.3 (92.1–95.8)	2.3 (−0.7 to 5.3)
**Risk perception of getting infected** ^b^							
Probability in general	234	27.0 (24.1–30.0)	80	31.0 (25.7–36.9)	154	25.3 (22.0–28.9)	6.0 (−0.9 to 12.8)
In crowded closed spaces	699	80.6 (77.9–83.1)	217	84.1 (79.1–88.1)	482	79.1 (75.7–82.2)	5.9 (0.3 to 11.5)
In crowded open spaces	387	44.6 (41.4–48.0)	116	45.0 (39.0–51.1)	271	44.5 (40.6–48.5)	1.4 (−6.1 to 9.0)
In meetings with family and friends	511	58.9 (55.6–62.2)	157	60.9 (54.8–66.6)	354	58.1 (54.2–62.0)	2.2 (−5.2 to 9.6)
In on–site work	384	44.3 (41.0–47.6)	108	41.9 (36.0–48.0)	276	45.3 (41.4–49.3)	−4.0 (−11.5 to 3.5)
In public transport	628	72.4 (69.4–75.3)	188	72.9 (67.1–78.0)	440	72.2 (68.6–75.7)	0.0 (−6.8 to 6.8)
In education centers	311	35.9 (32.7–39.1)	74	28.7 (23.5–34.5)	237	38.9 (35.1–42.9)	−9.9 (−16.9 to −2.9)
In healthcare centers	387	44.6 (41.4–48.0)	112	43.4 (37.5–49.5)	275	45.2 (41.2–49.1)	−0.4 (−8.0 to 7.2)
Perceived COVID−19 severity if infected^c^	278	32.1 (29.0–35.3)	79	30.6 (25.3–36.5)	199	32.7 (29.1–36.5)	0.3 (−6.4 to 7.1)
**Worry about possible pandemic consequences** ^d^							
Losing a loved one	802	92.5 (90.5–94.1)	243	94.2 (90.6–96.5)	559	91.8 (89.3–93.7)	2.1 (−1.6 to 5.9)
Health services overload	786	90.7 (88.5–92.4)	234	90.7 (86.5–93.7)	552	90.6 (88.1–92.7)	1.1 (−3.2 to 5.3)
Losing their job	477	55.0 (51.7–58.3)	146	56.6 (50.5–62.5)	331	54.4 (50.4–58.3)	−1.4 (−8.9 to 6.0)
Inability to pay the bills	525	60.6 (57.3–63.8)	163	63.2 (57.1–68.9)	362	59.4 (55.5–63.3)	3.3 (−4.0 to 10.5)
Work and family conciliation problem	478	55.1 (51.8–58.4)	164	63.6 (57.5–69.2)	314	51.6 (47.6–55.5)	12.2 (4.8 to 19.5)
Closure of schools or education centers	427	49.3 (45.9–52.6)	171	66.3 (60.3–71.8)	256	42.0 (38.2–46.0)	26.5 (19.4 to 33.6)

a Frequency and percentage of correct answers.

b Frequency and percentage of answers “likely” or “very likely” (4 or 5 points over 5).

c Frequency and percentage of answers “severe” or “very severe” (4 or 5 points over 5).

d Frequency and percentage of being worried or very worried.

e Standardized to the overall distribution of sex, age, education, living with someone older than 60, and personal history of COVID-19 in the entire COSMO-SPAIN sample.

The general risk perception of getting infected, and about the severity of the COVID-19 in the case they got the disease was relatively low among adults, irrespective of the presence of children in the household. Participants assigned the highest risk of infection to crowded closed spaces, where 80.6% (95% CI: 77.9–83.1) of the overall sample considered infection to be likely or very likely, and this opinion was more prevalent among those living with children (SPrD: 5.9%; 95% CI: 0.3–11.5). Other places frequently considered as risky for getting infected were public transportation, where 72.4% (95% CI: 69.4–75.3) considered infection as likely or very likely, and meeting with family and relatives (58.9%; 95% CI: 55.6–62.2), with no differences between those living or not living with children. On the contrary, educative centers were perceived as safer places, mainly among those living with children (SPrD: −9.9%; 95% CI: −16.9 to −2.9).

Fear of losing a loved one and of the overloading of health services were the most important concerns among both, adults living with children and those not living with children. The most evident difference between these groups was the higher worry, among those living with children, about closure of education centers and about work and family conciliation (SPrD: 26.5 % (95% CI: 19.4–33.6), and 12.2% (95% CI: 4.8–19.5), respectively) (Table 4).

## 4. Discussion

Overall, our findings revealed that adults in general and, particularly those living with children, have a good level of knowledge on COVID-19 transmission mechanisms as well as the preventive measures to be followed. Living with children was associated to a slightly higher compliance with mask using and social distancing, with more avoidance of crowded spaces and bars, and a lower use of public transport. Also, this subgroup had specific worries related with the difficulties of having children at home (i.e., work conciliation and school closures), while they mostly considered schools as a safe place, more than those without kids at home.

The observed high level of knowledge and compliance with preventive recommendations is reassuring, since parents are the primary protectors of their children' health, being responsible for their health education together with the role of the schools (10). Indeed, the health behavior of parents may influence the children's health, by increasing or decreasing the risk of transmission of the infection both, directly by influencing children' behavior, and indirectly by reducing their own risk of infection and consequently the transmission to their children. The better the parents are informed about the preventive measures to avoid the COVID-19, the better would be their adherence to them (20). Although other factors must also be taken into account, including those related to attitudes, risk perception, self-efficacy or personality characteristics (21, 22). According to COSMO-SPAIN data, there were very small differences in the knowledge on SARS-CoV-2 transmission mechanisms and correct use of preventive measures between participants living or not with children. Only the items about timing of symptoms onset, mandatory nature of recommendations, washing hands when using a mask and efficacy of physical distance were slightly more correctly answered by people living with children (standardized differences around 2%, but with wide confidence intervals). The higher belief among this group that physical distance is an effective measure is consistent with their slightly higher compliance with the recommendation of maintaining physical distance. However, in general, the similarity in knowledge between both groups suggests that the differences observed in compliance with preventive measures would be mainly led by factors other than knowledge.

The lower risk of getting infected in education centers perceived by people living with children, compared to those living in households without children, could be related to higher knowledge of the strict control measures adopted in this environment (23), as well as to the relatively few cases identified in the outbreaks occurred in this setting since the reopening of schools in Spain in September 2020 (24–26). This supports the idea that personal experience modulates the beliefs and perceptions that the population acquires from other sources such as the media or health authorities (27, 28) and suggests that parents trusted the risk-reduction strategies adopted by health and educative authorities at that time (i.e., compulsory mask and distance at school; students' groups split into two that attended school on alternate days; entrance through different doors and at different times; or combination of onsite and online teaching).

In both studies, COSMO-SPAIN and ENE-COVID, always wearing a mask in gatherings with family or friends was much less frequent than in other settings or in general, irrespective of living in a household with or without children. This could be explained by the lower perception of risk of infection in this setting compared to public transports or crowded closed places, and to social pressure not to follow this recommendation in the familiar environment (29). Maintaining the recommended physical distance was also scarcely followed, according to COSMO-SPAIN results. As our definition of compliance implied answering that the measure was followed “always”, this low compliance may reflect the impossibility of maintaining physical distance in certain situations, e.g. in public transports or when it depends on the behavior of other people, more than a lack of commitment with compliance (29). This would be congruent with the much higher compliance found at work in the ENE-COVID study, a setting where structural and organizational measures were implemented to separate people, whenever it was feasible.

Consistently with their perception of high risk of infection in closed environments, such as bars, indoor restaurants, public transport and, to a lesser extent, during family meetings, we found that the use of masks was very frequent during displacements and during leisure time activities. Also, the higher perception of contagion risk in crowded closed spaces among those living with children was in line with the finding that they went less frequently to bars and used less the public transportation. These results are in agreement with previous studies carried out during the current COVID-19 pandemic (20) and during previous flu pandemics (30) that show that knowledge and risk perception may be key predictors of the compliance with preventive measures. However, given the cross-sectional approach of our analysis, we cannot rule out that these differences in behaviors were related to factors associated with the fact of living with children other than the different risk perception or the desire to protect kids. For instance, people living with children may have lower opportunities to socialize in bars, or more difficulties with using public transport.

A possible influence of living with children in health-related behaviors among adults has been previously reported, although results from different studies are not fully in agreement. Some authors reported healthier behaviors among people living with children, while others found no differences or opposite relations (31). Regarding preventive measures against communicable diseases, some publications reported lower COVID-19 vaccine hesitancy or higher vaccine uptake among people living with children (32, 33) as well as higher social interaction reduction among rural females living with children compared with those not living with children (34). Although small in magnitude, our findings, adjusted by potential confounding factors such as age, education, sex, living with someone older than 60 and personal history of COVID-19, would add support for a positive association between the presence of children in households and a better adherence of adults to preventive measures related to infectious diseases. A possible explanation for these differences could be that the desire of protecting children would encourage those living with them to follow more strictly the recommendations. According to the Health Belief Model, the perceived susceptibility to acquire a disease and perceived disease severity are two of the factors that influence the adoption of preventive behaviors (35, 36). Although COVID-19 has been found to be usually mild in children, the mere possibility that it could be severe may be perceived by parents as a terrible consequence and lead them to reinforce their compliance with precautions. The perception of a higher probability of getting infected, or the higher worry about restrictions such as closure of schools if epidemiological situation worsened, as were estimated from the COSMO-SPAIN data among participants living with children, could also have contributed to the slightly higher compliance observed in this group.

Adults living with children reported being worried or very worried about work and family conciliation more frequently than those not living with children. This was also reflected in their higher concern about schools closure. This last worry was probably also related to concerns about education quality. Indeed, surveys that estimate parents' concern regarding their children's education, showed more concern when their children were remote learners, perhaps partly due to the higher implication of parents in academic education it involved (37, 38), a task for which not all of them felt well prepared. Lack of the necessary technological resources for the online education could also explain this finding. Considering the importance of this issue (more than 60% of COSMO-SPAIN participants were worried or very worried about it), both educators and policymakers face an important challenge to find effective strategies to improve the child learning experience during this singular situation (39, 40) and to avoid increasing inequity. Achieving these goals would have a positive impact, not only in children, but also in their parents, by alleviating the increased stress parents may suffer from this situation, especially if they have jobs with higher conciliation difficulties and if no changes in workplace policies have been made to protect workers with dependent children (41).

Some aspects of this study can be highlighted as its main strengths. Firstly, we used data from two nationwide studies with information on behavior and about KAP, which have not been extensively studied in such big samples. The study designs made it possible to analyze differences between two population groups (based on the presence or absence of children in the household) regarding the compliance with preventive measures facing SARS-CoV-2 infection, and to identify that there were some specific concerns related to the pandemic in each of these groups. Secondly, all data were collected during the same period and therefore explore the same epidemiological context and containment measures. This allowed us to compare the information and to do a parallel analysis. Both surveys complement each other with study-specific information derived from their different focus, i.e., a biomedical and behavioral focus in the ENE-COVID, and a psychosocial focus in COSMO-SPAIN. In both studies the participation rate was high. Also, the sampling design of the ENE-COVID survey, together with statistical analyses that took into account sampling weights and differential non-response, make its results representative of the Spanish non-institutionalized population.

Yet, our study has also several limitations. Comparison between both studies is hampered by their different sample selection and data collection methods (i.e., random selection and face-to-face or telephone interviews in ENE-COVID, panel data and online survey in COSMO-SPAIN). The higher proportion of participants in the youngest age group and with university education in the COSMO-SPAIN compared to the ENE-COVID study may be due to these differences. This also may imply a variation in the validity of the answers collected, and different selection biases may appear: in the case of online self-administered questionnaires such as the COSMO-SPAIN study, they require internet literacy. In the case of interview-administered questionnaires, the presence of the interviewer may help to reduce the uncertainty that respondents may experience with some items, thereby increasing the validity of the results (42), but also may increase the risk of social desirability bias. In this sense, the fact that information on behaviors was self-reported, may have led to some overestimation of the degree of compliance. Also, the differences observed between groups could be overestimated if people living with children were more prone to the social desirability bias. Although we are not aware of any evidence on a differential tendency to this bias depending on the presence of children in the household, parents could be more susceptible to it, given that they are supposed to act as a model for their children. On the other hand, in ENE-COVID, a large number of professionals were involved in data collection, which may have introduced variability in the process, in spite of the shared training platform established and the monitoring of the data collection. Lastly, although we carefully selected the questions that were similar enough in the respective questionnaires to be compared between studies, their wording and time frame were not identical and, therefore, they could have some different nuances that limits their comparability.

Our findings reinforce the need for providing support to parents and children to adapt to children distance learning and to coordinate work and family life. In order to improve COVID-19 related knowledge, a better communication about timing of symptoms' onset once infection has been acquired would be useful, since this was one of the worst answered items both in participants with and without children. Reinforcing the importance of wearing a mask also in gatherings with family and friends, again in both groups, could contribute to improve control of SARS-CoV-2 transmission.

## 5. Conclusion

The juxtaposition of the results of the COSMO-SPAIN and the ENE-COVID surveys showed that during the second wave of the pandemic in Spain, young and middle-aged adults living with children adopted some social distancing measures to prevent the transmission of SARS-CoV-2 slightly more frequently than those living without children. Putting the results of these two studies together highlighted the consistency of the information provided by them. It also showed that different research approaches can be able to provide complementary answers to a common public health issue and make the understanding of the problem more complete, which could aid in the formulation of more precise public health recommendations.

## Data availability statement

The datasets presented in this article are not readily available because of privacy issues, but COSMO-SPAIN data is available from the authors upon reasonable request, and ENE-COVID data will be available under approval by a Scientific Board that will evaluate the petitions and guarantee the safeguard of participants' rights, under the limits imposed by the Ethics Committee. Requests to access the datasets should be directed to cosmo-spain@isciii.es and to follow the procedure at the study webpage https://portalcne.isciii.es/enecovid19.

## Ethics statement

The studies involving human participants were reviewed and approved by the Research Ethics Committee of the Instituto de Salud Carlos III. Written informed consent to participate in this study was provided by the participants or the participants' legal guardian/next of kin.

## ENE-COVID study group

Spanish Ministry of Health: Pilar Aparicio Azcárraga; Faustino Blanco; Rodrigo Gutiérrez Fernández; Mariano Martín; Saturnino Mezcua Navarro; Marta Molina; Juan F. Muñoz-Montalvo; Matías Salinero Hernández; Jose L. Sanmartín. Institute of Health Carlos III: Manuel Cuenca-Estrella; José León Paniagua; Raquel Yotti; National Center of Epidemiology: Nerea Fernández de Larrea Baz; Pablo Fernández-Navarro; Roberto Pastor-Barriuso; Beatriz Pérez-Gómez; Marina Pollán; National Center of Microbiology: Ana Avellón; Giovanni Fedele; Aurora Fernández-García; Jesús Oteo Iglesias; María Teresa Pérez Olmeda; National School of Public Health: Israel Cruz; Maria Elena Fernández Martínez; Francisco D. Rodríguez-Cabrera. Harvard T.H. Chan School of Public Health: Miguel A. Hernán. Spanish Regional Health Services: Andalucía: José M. Navarro Marí; Susana Padrones Fernández; Begoña Palop Borrás; Ana Belén Pérez Jiménez; Manuel Rodríguez-Iglesias; José Manuel Rumbao Aguirre. Aragón: Ana María Calvo Gascón; María Luz Lou Alcaine. Asturias: Ignacio Donate Suárez; Mercedes Rodríguez Pérez; Oscar Suárez Álvarez. Baleares: Lluis Carbo Saladrigas; Margarita Cases Sanchís; Adoración Hurtado Fernández; Antonio Oliver; Carlos Javier Villafáfila Gomila. Canarias: José María Barrasa Fernández; Elías Castro Feliciano; María Noemí González Quintana; María Araceli Hernández Betancor; Melisa Hernández Febles; Leopoldo Martín Martín. Cantabria: Inés De Benito Población; Luis-Mariano López López; Teresa Ugarte Miota. Castilla-La Mancha: María Sagrario Celada Pérez; María Natalia Vallés Fernández. Castilla y León: Marta Domínguez-Gil González; Isabel Fernández-Natal; Tomás Maté Enríquez; Gregoria Megías Lobón; Juan Luis Muñoz Bellido; Miguel Villa Arranz. Cataluña: Pilar Ciruela; Maria Doladé Botías; M. Angeles Marcos Maeso; Ariadna Mas i Casals; Dúnia Pérez del Campo. Comunidad Valenciana: Antonio Félix de Castro; Ramón Limón Ramírez. Extremadura: Maria Francisca Elías Retamosa; Manuela Rubio González. Galicia: Antonio Aguilera; María Sinda Blanco Lobeiras; German Bou; Alberto Fuentes Losada. La Rioja: Yolanda Caro; Noemí Marauri; Luis Miguel Soria Blanco. Madrid: Roberto Alonso Fernández; Isabel del Cura González; Montserrat Hernández Pascual; Paloma Merino-Amador. Murcia: Natalia Cabrera Castro; Cristóbal Ramírez Almagro; Manuel Segovia Hernández; Aurora Tomás Lizcano. Navarra: Nieves Ascunce Elizaga; María Ederra Sanz; Carmen Ezpeleta Baquedano. País Vasco: Ana Bustinduy Bascaran; Luis Elorduy Otazua; Susana Iglesias Tamayo. Ceuta: Rebeca Benarroch Benarroch; Jesús Lopera Flores. Melilla: Antonia Vázquez de la Villa.

## Author contributions

IJ, MF, BP-G, and RP-B were responsible for the conceptualization and design of this study. IJ and NF performed the statistical analysis, developed the tables and figures design, and drafted all manuscript versions. IJ, CR-B, RP-B, MF, MP-S, and BP-G contributed to interpretation of data. All authors have contributed to reviewing and editing the last version of the manuscript.
