# Supplementary material for: Preventive behavior against SARS-CoV-2 infection in adults according to whether or not they live with children. A combined analysis of the nationwide COSMO-SPAIN and ENE-COVID surveys
Source: Front Public Health. 2023 Feb 23;11:1061367. doi: 10.3389/fpubh.2023.1061367 (PMC9996036; doi:10.3389/fpubh.2023.1061367)
Supplement: Supplementary file 1 [file Data_Sheet_1.PDF]

Supplementary Material

Supplementary Table 1. Adherence to COVID-19 preventive recommendations in the COSMO-SPAIN survey, according to sociodemographic characteristics and COVID-19 experience

	Mask use					Maintaining physical distance in general	Social distance		Ventilating closed spaces in general	Avoid public transport
	Always, in general	Always in family meetings/with friends	Mask type				Avoiding meeting with family	Avoiding crowded places		
			Hygienic	Surgical	FFP2					
	n (%)	n (%)	n (%)	n (%)	n (%)	n (%)	n (%)	n (%)	n (%)	n (%)
**Sex**										
Men	309 (75.9)	249 (61.2)	75 (20.8)	178 (49.3)	108 (29.9)	166 (40.8)	144 (35.4)	211 (51.8)	244 (60.0)	265 (65.1)
Women	378 (82.2)	283 (61.5)	82 (20.2)	202 (49.8)	122 (30.0)	198 (43.0)	209 (45.4)	249 (54.1)	319 (69.3)	282 (61.3)
**Age group (years)**										
24-34	164 (75.6)	101 (46.5)	36 (19.6)	97 (52.7)	51 (27.7)	70 (32.3)	59 (27.2)	95 (43.8)	119 (54.8)	136 (62.7)
35-44	174 (75.7)	133 (57.8)	42 (20.5)	100 (48.8)	63 (30.7)	95 (41.3)	79 (34.3)	119 (51.7)	139 (60.4)	143 (62.2)
45-54	201 (83.1)	165 (68.2)	49 (22.5)	103 (47.3)	66 (30.3)	107 (44.2)	124 (51.2)	137 (56.6)	178 (73.6)	164 (67.8)
55-64	148 (83.2)	133 (74.7)	30 (18.8)	80 (50.0)	50 (31.3)	92 (51.7)	91 (51.1)	109 (61.2)	127 (71.3)	104 (58.4)
**Education**										
Primary or less	161 (79.7)	139 (68.8)	31 (17.8)	105 (60.3)	38 (21.8)	103 (51.0)	93 (46.0)	109 (54.0)	137 (67.8)	121 (59.9)
Secondary	224 (81.2)	171 (62.0)	64 (26.1)	116 (47.3)	65 (26.5)	113 (40.9)	107 (38.8)	149 (54.0)	183 (66.3)	176 (63.8)
University	302 (77.6)	222 (57.1)	62 (17.8)	159 (45.7)	127 (36.5)	148 (38.0)	153 (39.3)	202 (51.9)	243 (62.5)	250 (64.3)
**Number of people living in the house**										
1	76 (77.6)	54 (55.1)	19 (22.1)	35 (40.7)	32 (37.2)	41 (41.8)	31 (31.6)	47 (48.0)	57 (58.2)	56 (57.1)
2	216 (78.6)	166 (60.4)	50 (20.6)	125 (51.4)	68 (28.0)	116 (42.2)	111 (40.4)	142 (51.6)	182 (66.2)	173 (62.9)
3	178 (82.4)	128 (59.3)	42 (21.8)	90 (46.6)	61 (31.6)	91 (42.1)	84 (38.9)	117 (54.2)	145 (67.1)	130 (60.2)
4	143 (77.3)	123 (66.5)	33 (20.1)	86 (52.4)	45 (27.4)	74 (40.0)	87 (47.0)	105 (56.8)	124 (67.0)	130 (70.3)
>=5	74 (79.6)	61 (65.6)	13 (16.0)	44 (54.3)	24 (29.6)	42 (45.2)	40 (43.0)	49 (52.7)	55 (59.1)	58 (62.4)
**Living with older than 60**										
No	548 (78.4)	420 (60.1)	124 (20.2)	304 (49.5)	186 (30.3)	297 (42.5)	279 (39.9)	367 (52.5)	450 (64.4)	444 (63.5)
Yes	139 (82.7)	112 (66.7)	33 (21.6)	76 (49.7)	44 (28.8)	67 (39.9)	74 (44.0)	93 (55.4)	113 (67.3)	103 (61.3)
**Employment**										
No	243 (81.0)	191 (63.7)	55 (21.4)	136 (52.9)	66 (25.7)	140 (46.7)	131 (43.7)	171 (57.0)	203 (67.7)	193 (64.3)
Yes	444 (78.3)	341 (60.1)	102 (20.0)	244 (47.8)	164 (32.2)	224 (39.5)	222 (39.2)	289 (51.0)	360 (63.5)	354 (62.4)

(Table continues)

Supplementary Table 1 (continued)

	Mask use					Maintaining physical distance in general	Social distance		Ventilating closed spaces in general	Avoid public transport
	Always, in general	Always in family meetings/with friends	Mask type				Avoiding meeting with family	Avoiding crowded places		
			Hygienic	Surgical	FFP2					
	n (%)	n (%)	n (%)	n (%)	n (%)	n (%)	n (%)	n (%)	n (%)	n (%)
**Healthcare worker[a]**										
No	425 (77.8)	326 (59.7)	101 (20.6)	234 (47.8)	154 (31.5)	216 (39.6)	213 (39.0)	281 (51.5)	346 (63.4)	341 (62.5)
Yes	19 (90.5)	15 (71.4)	1 (4.8)	10 (47.6)	10 (47.6)	8 (38.1)	9 (42.9)	8 (38.1)	14 (66.7)	13 (61.9)
**Telework***										
No	366 (78.5)	290 (62.2)	76 (18.3)	197 (47.5)	142 (34.2)	189 (40.6)	186 (39.9)	236 (50.6)	299 (64.2)	283 (60.7)
Yes	78 (77.2)	51 (50.5)	26 (27.4)	47 (49.5)	22 (23.2)	35 (34.7)	36 (35.6)	53 (52.5)	61 (60.4)	71 (70.3)
**Regional seroprevalence**										
<6.5%	316 (79.6)	241 (60.7)	73 (20.6)	181 (51.1)	100 (28.3)	174 (43.8)	164 (41.3)	211 (53.2)	267 (67.3)	269 (67.8)
[6.5-9)%	54 (75.0)	51 (70.8)	13 (20.3)	32 (50.0)	19 (29.7)	24 (33.3)	32 (44.4)	34 (47.2)	52 (72.2)	36 (50.0)
[9-12)%	163 (83.2)	120 (61.2)	33 (19.2)	85 (49.4)	54 (31.4)	80 (40.8)	91 (46.4)	109 (55.6)	126 (64.3)	111 (56.6)
>=12%	154 (76.2)	120 (59.4)	38 (21.5)	82 (46.3)	57 (32.2)	86 (42.6)	66 (32.7)	106 (52.5)	118 (58.4)	131 (64.8)
**Personal history of COVID-19**										
No infection	629 (78.8)	493 (61.8)	143 (20.3)	350 (49.7)	212 (30.1)	342 (42.9)	330 (41.3)	428 (53.6)	515 (64.5)	509 (63.8)
Not severe infection	54 (88.5)	35 (57.4)	12 (21.8)	29 (52.7)	14 (25.5)	19 (31.1)	20 (32.8)	27 (44.3)	42 (68.8)	33 (54.1)
Pneumonia or hospitalization	4 (50.0)	4 (50.0)	2 (28.6)	1 (14.3)	4 (57.1)	3 (37.5)	3 (37.5)	5 (62.5)	6 (75.0)	5 (62.5)
**Test confirmed self-reported COVID-19**										
No	644 (78.7)	501 (61.3)	149 (20.7)	353 (49.0)	218 (30.3)	347 (42.4)	333 (40.7)	433 (52.9)	528 (64.6)	519 (63.5)
Yes	43 (87.8)	31 (63.3)	8 (17.0)	27 (57.5)	12 (25.5)	17 (34.7)	20 (40.8)	27 (55.1)	35 (71.4)	28 (57.1)
**A close friend or relative had COVID-19**										
No	283 (80.2)	227 (64.3)	59 (19.2)	165 (53.6)	84 (27.3)	186 (52.7)	158 (44.8)	194 (55.0)	242 (68.6)	237 (67.1)
Yes	404 (78.6)	305 (59.3)	98 (21.4)	215 (46.8)	146 (31.8)	178 (34.6)	195 (37.9)	266 (51.8)	321 (62.5)	310 (60.3)

[a] Among employed participants.

Supplementary Table 2. Adherence to COVID-19 preventive recommendations in the ENE-COVID survey, according to sociodemographic characteristics and COVID-19 experience.

	Mask use							Maintain physical distance at work	Ventilating in the workplace	Social distance				Not using public transport
	Always in family meetings/with friends	Always at work	Always during leisure	Always during displacements	Mask type					Avoid family meetings	Avoid social events with >10 people	Avoid going to bars, inside	Avoid going to bars, outside	
					Hygienic	Surgical	FFP2							
	n (%)	n (%)	n (%)	n (%)	n (%)	n (%)	n (%)	n (%)	n (%)	n (%)	n (%)	n (%)	n (%)	n (%)
**Sex**														
Men	5,411	9,037	13,103	13,419	2,718	8,466	2,634	8,788	9,449	1,911	9,790	8,729	4,652	10,967
	(45.4)	(88.9)	(96.8)	(97.7)	(19.7)	(61.3)	(19.1)	(86.4)	(92.9)	(13.8)	(70.8)	(63.1)	(33.6)	(79.3)
Women	6,465	9,421	15,350	15,777	2,650	9,924	3,500	8,140	9,167	2,414	11,726	12,112	6,435	12,109
	(47.3)	(93.5)	(97.5)	(98.7)	(16.5)	(61.7)	(21.8)	(80.8)	(91.0)	(15.0)	(72.8)	(75.3)	(40.0)	(75.2)
**Age group (years)**														
25-34	1,600	2,959	4,097	4,212	887	2,547	873	2,541	2,856	379	2,743	2,725	1,209	3,320
	(40.7)	(93.1)	(96.2)	(98.2)	(20.6)	(59.1)	(20.3)	(80.0)	(89.9)	(8.8)	(63.6)	(63.2)	(28.0)	(77.0)
35-44	2,813	5,268	7,106	7,257	1,534	4,266	1,619	4,703	5,191	924	5,003	5,236	2,629	5,881
	(43.3)	(92.8)	(97.3)	(98.3)	(20.7)	(57.5)	(21.8)	(82.8)	(91.4)	(12.4)	(67.4)	(70.5)	(35.4)	(79.2)
45-54	3,891	6,070	8,767	8,998	1,682	5,672	1,843	5,647	6,169	1,437	6,681	6,377	3,407	7,044
	(50.1)	(90.8)	(97.4)	(98.3)	(18.3)	(61.7)	(20.0)	(84.5)	(92.3)	(15.6)	(72.5)	(69.2)	(37.0)	(76.5)
55-64	3,572	4,161	8,483	8,729	1,265	5,905	1,799	4,037	4,400	1,585	7,089	6,503	3,842	6,831
	(48.3)	(88.6)	(97.2)	(98.0)	(14.1)	(65.8)	(20.1)	(85.9)	(93.7)	(17.6)	(78.9)	(72.4)	(42.8)	(76.1)
**Education level**														
Primary	4,538	5,495	10,349	10,637	2,006	7,285	1,651	5,252	5,841	1,655	8,397	7,865	5,062	8,217
or less	(48.8)	(88.0)	(97.1)	(97.9)	(18.3)	(66.6)	(15.1)	(84.1)	(93.6)	(15.1)	(76.7)	(71.8)	(46.2)	(75.0)
	4,077	6,966	9,999	10,231	1,970	6,333	2,178	6,286	6,883	1,581	7,565	7,256	3,681	8,198
Secondary	(45.8)	(92.3)	(97.3)	(98.2)	(18.8)	(60.4)	(20.8)	(83.3)	(91.2)	(15.1)	(72.1)	(69.2)	(35.1)	(78.1)
	3,165	5,823	7,840	8,045	1,336	4,589	2,257	5,213	5,708	1,038	5,333	5,523	2,224	6,441
University	(44.2)	(93.0)	(97.0)	(98.6)	(16.3)	(56.1)	(27.6)	(83.3)	(91.2)	(12.7)	(65.1)	(67.4)	(27.1)	(78.6)
**Number of people living in the house**														
1	763	1,205	1,801	1,858	288	1,231	390	1,111	1,216	263	1,456	1,256	610	1,384
	(46.3)	(90.8)	(97.1)	(97.7)	(15.1)	(64.5)	(20.4)	(83.7)	(91.6)	(13.8)	(76.2)	(65.8)	(31.9)	(72.5)
2	2,775	4,003	6,640	6,849	1,124	4,313	1,579	3,622	4,050	1,066	5,333	4,899	2,593	5,283
	(46.5)	(90.9)	(96.8)	(98.1)	(16.0)	(61.5)	(22.5)	(82.3)	(92.0)	(15.2)	(75.9)	(69.7)	(36.9)	(75.2)
3	3,362	5,201	8,081	8,290	1,500	5,269	1,699	4,771	5,297	1,225	6,158	5,897	3,142	6,585
	(46.4)	(90.3)	(97.3)	(98.5)	(17.7)	(62.2)	(20.1)	(82.9)	(92.0)	(14.5)	(72.7)	(69.6)	(37.1)	(77.7)
4	3,381	5,562	8,031	8,231	1,646	5,060	1,697	5,104	5,546	1,197	5,731	5,824	3,022	6,677
	(46.8)	(92.1)	(97.3)	(98.3)	(19.6)	(60.2)	(20.2)	(84.5)	(91.8)	(14.2)	(68.1)	(69.2)	(35.9)	(79.3)
≥5	1,595	2,487	3,900	3,968	810	2,517	769	2,320	2,507	574	2,838	2,965	1,720	3,147
	(45.2)	(91.7)	(97.2)	(97.7)	(19.8)	(61.5)	(18.8)	(85.6)	(92.4)	(14.0)	(69.2)	(72.3)	(42.0)	(76.8)

(Table continues)

Supplementary Table 2 (continued)

	Mask use							Maintain physical distance at work	Ventilating in the workplace	Social distance				Not using public transport
	Always in family meetings/with friends	Always at work	Always during leisure	Always during displacements	Mask type					Avoid family meetings	Avoid social events with >10 people	Avoid going to bars, inside	Avoid going to bars, outside	
					Hygienic	Surgical	FFP2							
	n (%)	n (%)	n (%)	n (%)	n (%)	n (%)	n (%)	n (%)	n (%)	n (%)	n (%)	n (%)	n (%)	n (%)
**Living with older than 60**														
No	8,429 (46.1)	13,843 (91.6)	20,236 (97.2)	20,800 (98.3)	3,955 (18.6)	13,070 (61.5)	4,227 (19.9)	12,623 (83.5)	13,873 (91.8)	3,006 (14.1)	14,969 (70.4)	14,683 (69.0)	7,543 (35.5)	16,509 (77.6)
Yes	3,445 (47.0)	4,613 (90.0)	8,215 (97.1)	8,394 (98.1)	1,413 (16.4)	5,318 (61.6)	1,907 (22.1)	4,303 (84.0)	4,742 (92.5)	1,319 (15.3)	6,545 (75.7)	6,156 (71.2)	3,542 (41.0)	6,567 (75.9)
**Employment**														
No	4,129 (49.2)	1,734 (92.7)	9,368 (97.2)	9,629 (98.1)	1,849 (18.7)	6,398 (64.7)	1,645 (16.6)	1,544 (82.6)	1,721 (92.0)	1,503 (15.2)	7,587 (76.6)	7,452 (75.3)	4,463 (45.1)	7,452 (75.3)
Yes	7,747 (45.0)	16,724 (91.0)	19,085 (97.1)	19,567 (98.2)	3,519 (17.6)	11,992 (60.0)	4,489 (22.5)	15,384 (83.7)	16,895 (92.0)	2,822 (14.1)	13,929 (69.6)	13,389 (66.9)	6,624 (33.1)	15,624 (78.0)
**Health care worker**[a]														
No	7,258 (44.8)	15,597 (90.5)	17,992 (97.1)	18,446 (98.2)	3,476 (18.4)	11,280 (59.8)	4,106 (21.8)	14,601 (84.7)	15,902 (92.3)	2,683 (14.2)	13,132 (69.5)	12,579 (66.6)	6,260 (33.2)	14,726 (78.0)
Yes	489 (48.9)	1,127 (98.9)	1,093 (97.3)	1,121 (98.7)	43 (3.8)	712 (62.6)	383 (33.7)	783 (68.7)	993 (87.2)	139 (12.2)	797 (70.0)	810 (71.1)	364 (32.0)	898 (78.8)
**Telework***														
No	7,002 (44.4)	16,724 (91.0)	17,504 (97.1)	17,954 (98.3)	3,181 (17.3)	11,014 (60.0)	4,153 (22.6)	15,384 (83.7)	16,895 (92.0)	2,617 (14.2)	12,807 (69.7)	12,250 (66.7)	6,049 (32.9)	14,354 (78.1)
Yes	745 (51.5)	NA	1,581 (97.1)	1,613 (98.2)	338 (20.5)	978 (59.2)	336 (20.3)	NA	NA	205 (12.4)	1,122 (67.9)	1,139 (68.9)	575 (34.8)	1,270 (76.9)
**Municipality size (habitants)**														
>100,000	3,347 (43.7)	5,653 (92.3)	8,524 (96.8)	8,850 (98.8)	1,714 (19.1)	5,332 (59.3)	1,949 (21.7)	5,044 (82.3)	5,515 (90.0)	1,343 (14.9)	6,481 (72.0)	6,223 (69.1)	2,873 (31.9)	6,085 (67.6)
20,000-100,000	3,629 (46.8)	5,531 (93.1)	8,548 (97.2)	8,806 (98.6)	1,524 (17.0)	5,661 (63.1)	1,783 (19.9)	5,052 (85.1)	5,476 (92.2)	1,213 (13.5)	6,450 (71.9)	6,375 (71.1)	3,355 (37.4)	7,193 (80.2)
5,000-20,000	2,555 (46.5)	4,045 (90.7)	6,206 (97.5)	6,335 (97.8)	1,244 (19.1)	3,945 (60.6)	1,319 (20.3)	3,684 (82.6)	4,124 (92.4)	1,014 (15.6)	4,685 (71.9)	4,604 (70.7)	2,535 (38.9)	5,304 (81.4)
<5,000	2,345 (50.1)	3,229 (86.9)	5,175 (97.1)	5,205 (96.9)	886 (16.3)	3,452 (63.7)	1,083 (20.0)	3,148 (84.8)	3,501 (94.3)	755 (13.9)	3,900 (71.8)	3,639 (67.0)	2,324 (42.8)	4,494 (82.7)

(Table continues)

Supplementary Table 2 (continued)

	Mask use									Social distance				
					Mask type									
	Always in family meetings/with friends	Always at work	Always during leisure	Always during displacements	Hygienic	Surgical	FFP2	Maintain physical distance at work	Ventilating in the workplace	Avoid family meetings	Avoid social events with >10 people	Avoid going to bars, inside	Avoid going to bars, outside	Not using public transport
	n (%)	n (%)	n (%)	n (%)	n (%)	n (%)	n (%)	n (%)	n (%)	n (%)	n (%)	n (%)	n (%)	n (%)
**Census section average income (euros)**														
> 17,000	577	1,008	1,531	1,580	313	890	407	968	994	222	1,076	1,046	396	1,187
	(41.5)	(89.8)	(96.0)	(98.3)	(19.4)	(55.3)	(25.3)	(86.2)	(88.5)	(13.8)	(66.7)	(64.8)	(24.5)	(73.6)
12,500-17,000	2,370	4,200	6,144	6,265	1,238	3,777	1,411	3,773	4,145	979	4,543	4,362	1,943	4,917
	(43.5)	(91.6)	(97.3)	(98.0)	(19.3)	(58.8)	(22.0)	(82.3)	(90.4)	(15.2)	(70.6)	(67.8)	(30.2)	(76.4)
9,000-12,500	5,721	9,134	13,737	14,121	2,645	8,901	2,914	8,269	9,216	2,196	10,520	10,073	5,470	11,376
	(46.6)	(91.3)	(97.0)	(98.1)	(18.3)	(61.6)	(20.1)	(82.7)	(92.2)	(15.2)	(72.7)	(69.6)	(37.8)	(78.6)
7,700-9,000	1,923	2,696	4,425	4,537	741	3,016	904	2,542	2,776	627	3,398	3,305	1,998	3,566
	(47.6)	(90.9)	(97.3)	(98.3)	(15.9)	(64.7)	(19.4)	(85.7)	(93.6)	(13.4)	(72.8)	(70.8)	(42.8)	(76.4)
7,100-7,700	618	799	1,340	1,376	213	887	304	758	828	174	1,004	1,052	607	1,084
	(50.2)	(90.5)	(97.6)	(98.9)	(15.2)	(63.2)	(21.6)	(85.8)	(93.8)	(12.4)	(71.5)	(74.9)	(43.2)	(77.2)
< 7,100	667	621	1,276	1,317	218	919	194	618	657	127	975	1,003	673	946
	(55.3)	(90.5)	(97.6)	(99.0)	(16.4)	(69.1)	(14.6)	(90.1)	(95.8)	(9.5)	(73.2)	(75.3)	(50.5)	(71.0)
**Regional seroprevalence**														
<6.5%	2,870	5,117	7,868	8,144	1,515	5,222	1,642	4,643	5,137	1,270	6,091	5,662	3,020	6,547
	(40.3)	(91.2)	(96.8)	(97.7)	(18.1)	(62.3)	(19.6)	(82.8)	(91.6)	(15.1)	(72.6)	(67.5)	(36.0)	(78.1)
6.5-9%	3,394	4,955	7,725	7,973	1,289	5,088	1,728	4,503	5,026	1,101	5,686	5,958	3,028	6,133
	(48.4)	(91.9)	(96.8)	(98.9)	(15.9)	(62.8)	(21.3)	(83.5)	(93.2)	(13.6)	(70.1)	(73.4)	(37.3)	(75.6)
9-12%	1,907	3,167	4,859	4,978	1,199	2,880	1,015	2,965	3,189	804	3,825	3,722	2,090	3,837
	(44.4)	(91.2)	(97.4)	(98.5)	(23.5)	(56.5)	(19.9)	(85.4)	(91.8)	(15.8)	(75.0)	(73.0)	(41.0)	(75.3)
>=12%	3,705	5,219	8,001	8,101	1,365	5,200	1,749	4,817	5,264	1,150	5,914	5,499	2,949	6,559
	(51.6)	(90.5)	(97.5)	(97.9)	(16.4)	(62.5)	(21.0)	(83.5)	(91.3)	(13.8)	(71.0)	(66.0)	(35.4)	(78.8)
**History of COVID-19***														
No infection	10,106	15,738	24,385	25,044	4,661	15,624	5,352	14,487	15,882	3,727	18,487	17,965	9,526	19,829
	(46.1)	(91.1)	(97.1)	(98.2)	(18.2)	(60.9)	(20.9)	(83.9)	(91.9)	(14.5)	(72.0)	(70.0)	(37.1)	(77.3)
Not-severe infection	1,484	2,330	3,463	3,538	617	2,359	646	2,093	2,348	513	2,588	2,441	1,318	2,770
	(47.7)	(91.4)	(97.4)	(98.2)	(17.0)	(65.1)	(17.8)	(82.1)	(92.1)	(14.1)	(71.3)	(67.3)	(36.3)	(76.4)
Pneumonia/hospitalization	286	390	605	614	90	407	136	348	386	85	441	435	243	477
	(52.1)	(93.3)	(98.4)	(97.9)	(14.2)	(64.3)	(21.5)	(83.3)	(92.3)	(13.4)	(69.6)	(68.6)	(38.3)	(75.2)

(Table continues)

Supplementary Table 2 (continued)

	Mask use							Maintain physical distance at work	Ventilating in the workplace	Social distance				Not using public transport
	Always in family meetings/with friends	Always at work	Always during leisure	Always during displacements	Mask type					Avoid family meetings	Avoid social events with >10 people	Avoid going to bars, inside	Avoid going to bars, outside	
					Hygienic	Surgical	FFP2							
	n (%)	n (%)	n (%)	n (%)	n (%)	n (%)	n (%)	n (%)	n (%)	n (%)	n (%)	n (%)	n (%)	n (%)
**Self-reported positive PCR/antigen test**														
No	11,248 (46.1)	17,559 (91.1)	27,137 (97.1)	27,858 (98.2)	5,149 (18.0)	17,530 (61.5)	5,840 (20.5)	16,139 (83.7)	17,739 (92.1)	4,158 (14.6)	20,561 (72.0)	19,869 (69.6)	10,571 (37.0)	22,033 (77.2)
Yes	628 (52.0)	899 (92.7)	1,315 (98.0)	1,337 (97.6)	219 (16.0)	859 (62.6)	294 (21.4)	789 (81.3)	877 (90.4)	167 (12.1)	954 (69.4)	972 (70.7)	516 (37.5)	1,043 (75.9)
**Positive IgG in the Round 4**														
No	10,989 (46.3)	17,059 (91.2)	26,379 (97.1)	27,073 (98.2)	5,011 (18.1)	16,966 (61.2)	5,746 (20.7)	15,676 (83.8)	17,201 (91.9)	4,000 (14.4)	19,954 (71.9)	19,404 (69.9)	10,300 (37.1)	21,428 (77.2)
Yes	886 (48.0)	1,398 (91.1)	2,073 (97.6)	2,122 (98.4)	357 (16.5)	1,423 (65.6)	388 (17.9)	1,252 (81.6)	1,414 (92.2)	325 (15.0)	1,561 (71.9)	1,436 (66.1)	786 (36.2)	1,647 (75.8)
**Positive person in the house**														
No	10,202 (46.2)	15,843 (91.2)	24,508 (97.1)	25,166 (98.2)	4,615 (17.9)	15,767 (61.2)	5,389 (20.9)	14,553 (83.8)	15,970 (91.9)	3,738 (14.5)	18,569 (72.0)	18,068 (70.0)	9,611 (37.3)	19,919 (77.2)
Yes	1,674 (47.3)	2,615 (91.2)	3,945 (97.7)	4,030 (98.3)	753 (18.3)	2,623 (63.7)	745 (18.1)	2,375 (82.8)	2,646 (92.2)	587 (14.2)	2,947 (71.4)	2,773 (67.2)	1,476 (35.8)	3,157 (76.5)
**Contact with confirmed case last month**														
No	11,061 (46.6)	16,711 (90.8)	26,353 (97.2)	27,046 (98.2)	5,007 (18.1)	17,128 (61.8)	5,557 (20.1)	15,528 (84.4)	16,981 (92.3)	4,006 (14.4)	20,046 (72.3)	19,362 (69.8)	10,438 (37.7)	21,344 (77.0)
Yes	815 (43.3)	1,747 (94.9)	2,100 (96.9)	2,150 (98.0)	361 (16.4)	1,262 (57.4)	577 (26.2)	1,400 (76.1)	1,635 (88.9)	319 (14.5)	1,470 (66.7)	1,479 (67.1)	649 (29.5)	1,732 (78.6)

Supplementary Methods

Wording of questions about compliance with preventive measures included in the questionnaires of the COSMO-SPAIN and ENE-COVID surveys

COSMO-SPAIN

During the last seven days, how frequently did you take the following measures to avoid being infected by the coronavirus/COVID-19? (1- Never, ….. 5- Always):

- **Use mask following the recommendations** (corresponding variable in Table 3: *Always wearing face mask in general*)
- **Ventilate closed spaces** (corresponding variable in Table 3: *Ventilating closed spaces in general*)
- **Use mask when staying with friends** (corresponding variable in Table 3: *Always wearing face mask in family meetings/with friends*)
- **Not going to crowded places** (corresponding variable in Table 3: *Avoid crowded places*)
- **Avoid public transport** (corresponding variable in Table 3: *Avoid the use of public transport*)
- **Maintain physical distance (at least two meters)** (corresponding variable in Table 3: *Maintaining physical distance in general*)
- **Avoid social/family meetings** (corresponding variable in Table 3: *Avoid family meetings*)

What kind of mask do you usually use? Surgical mask; FFP2 mask; Hygienic mask; Other; Do not know (corresponding variable in Table 3: *Type of mask*. Options "Other" and "Do not know" were considered as missing).

ENE-COVID

Since the first of July 2020:

- **Have you usually use (at least once a week) public transport for your personal or working displacements?** Yes; No. (corresponding variable in Table 3: *Not using public transport*)
- **Do you wear a mask during your displacements, on foot or in public transport?** Yes; No; Sometimes; I didn't go out of home. (corresponding variable in Table 3: *Always wearing face mask during displacements*)
- **Do you wear a mask during meetings with family or friends (in your home or in theirs)?** Yes; No; Sometimes; I didn't gather. (corresponding variables in Table 3: *Always wearing face mask in family meetings/with friends*, and *Avoid family meetings*).
- **Do you wear a mask in other situations (shopping, leisure time, …) when you are with people that do not live with you?** Yes; No; Sometimes; I didn't meet other people. (corresponding variable in Table 3: *Always wearing face mask during leisure time*)
- **In general, what kind of mask do you use?** Cloth mask; Surgical mask; FFP2 mask. (corresponding variable in Table 3: *Type of mask*).
- **Have you gone out for a drink or for lunch on a terrace or outdoor bar?** No or sporadically (< once a month); From time to time (1-3 times/month); Some days (1-3 times/week); Almost every day (4-7 times/week). (corresponding variable in Table 3: *Avoid going outside bars*)
- **Have you gone out for a drink or for lunch on indoor bars or restaurants?** No or sporadically (< once a month); From time to time (1-3 times/month); Some days (1-3 times/week); Almost every day (4-7 times/week). (corresponding variable in Table 3: *Avoid going inside bars*)
- **How many times did you go to outdoor meetings, celebrations or activities with friends or relatives with more than 10 people?** Never; 1-5 times; 6-10 times; More than 10 times. (related variable* in Table 3: *Avoid social events with >10 people*)

- **How many times did you go to indoor meetings, celebrations or activities with friends or relatives (including at home), with more than 10 people?** Never; 1-5 times; 6-10 times; More than 10 times. (related variable* in Table 3: *Avoid social events with >10 people*)

For participants with on-site working/school activity:

Do you usually wear a mask in your workplace/school? Yes; No; Sometimes. (corresponding variable in Table 3: *Always wearing face mask in the working place*)

In general, in your workplace/school, are the following precautions followed?

- **Maintain a distance of at least 1.5 meters between people or there are physical barriers to separate them**: Yes; No. (corresponding variable in Table 3: *Maintaining physical distance at work*)
- **Ventilate the spaces frequently**: Yes; No. (corresponding variable in Table 3: *Ventilating working place*)

** The variable "Avoid social events with >10 people" was derived from the two questions related to frequency of attending meetings, celebrations or activities with friends and family. If neither outdoor nor indoor activities were done, it was considered that social events had been avoided; otherwise, this variable was coded as "No".*
